# Incidence and Management of Duodenal Trauma in a War Setting: Insights From a Military Hospital in Yemen

**DOI:** 10.7759/cureus.77323

**Published:** 2025-01-12

**Authors:** Yasser A Obadiel, Ali A Albrashi, Mohammed A Saeed, Haitham M Jowah

**Affiliations:** 1 Department of Surgery, Faculty of Medicine and Health Sciences, Sana'a University, Sana'a, YEM; 2 Department of Surgery, General Military Hospital, Sana'a, YEM

**Keywords:** duodenal injuries, exploratory laparotomy, injury severity score, postoperative complications, war trauma, yemen

## Abstract

Background

Duodenal injuries are rare but pose significant challenges in war trauma settings because of their complexity and associated complications. This study evaluated the incidence, anatomical distribution, surgical approaches, postoperative complications, and factors influencing the outcomes of duodenal injury management in a conflict setting in Yemen.

Methods

A retrospective analysis was conducted on 520 exploratory laparotomy cases from June 2019 to December 2023 at a military hospital in Yemen. Twenty-seven patients with confirmed duodenal injuries were included. Data on demographic characteristics, injury characteristics, surgical management, and outcomes were collected and analyzed.

Results

Among the 520 exploratory laparotomy cases reviewed, 27 (5.2%) patients had confirmed duodenal injuries. The study population was predominantly young males (n = 26, 96.3%) with a mean age of 21.93 ± 4.08 years. Penetrating trauma was the leading cause of injury (n = 26, 95.7%), and the second portion of the duodenum (D2) was the most frequently affected segment (n = 12, 44.4%). Most injuries were classified as the American Association for the Surgery of Trauma (AAST) grade II (n = 26, 96.3%). Surgical management primarily involved exploratory laparotomy (n = 22, 81.5%) and primary repair (n = 18, 66.7%). Postoperative complications occurred in 70.4% (n = 19), with sepsis (n = 10, 52.6%) and chest-related complications (n = 9, 47.4%) being the most common. The short-term success rate was 81.5% (n = 22), while the mortality rate was 11.1% (n = 3). Shrapnel injuries (80% vs. 13%, p = 0.009), higher injury severity scores (27.20 ± 9.34 vs. 19.14 ± 7.80, p = 0.05), and damage control surgery (60.0% vs. 9.1%, p = 0.008) were key factors associated with poorer outcomes.

Conclusion

Duodenal injuries after war trauma are associated with high rates of complications and mortality. Early diagnosis, appropriate surgical approaches, and vigilant postoperative care are critical for improved outcomes. These findings highlight the importance of tailored management strategies in conflict settings and the need for further research to optimize care protocols in resource-limited environments.

## Introduction

Duodenal injuries are a significant concern in war trauma because of their complexity and high risk of severe complications. Although their incidence in severely injured trauma patients ranges from 0.2% to 0.6%, they account for approximately 3% to 5% of abdominal trauma cases, with up to 80% resulting from penetrating injuries [1,2]. These injuries are often accompanied by vascular and adjacent organ damage, which is a significant complicating factor [3-5].

The management of duodenal injuries is highly complex, particularly in war zones, where unique challenges arise due to the severity of injuries and the demanding circumstances of trauma care during conflict. Mild-to-moderate injuries are typically treated with primary repair, while higher-grade injuries require advanced techniques such as duodenal diverticulization, pyloric exclusion, or pancreatoduodenectomy, all of which are associated with higher morbidity and mortality rates [6]. Postoperative complications, including sepsis, fistulas, and infections, occur in up to 60% of cases, with mortality rates ranging from 9.3% to 24% [7]. These outcomes emphasize the need for optimized management strategies and vigilant postoperative care to improve patient survival and recovery.

Despite advancements in surgical techniques, limited research has focused on duodenal injuries in war settings. Most studies have addressed general abdominal trauma, overlooking the unique challenges posed by war environments, such as delayed access to care, complex injury patterns, and resource constraints. Limited data hinder the development of standardized management protocols tailored to these scenarios.

In Yemen, ongoing conflict has resulted in a great burden of war-related trauma, including duodenal injuries [8]. However, data specific to the management and outcomes of such injuries are limited. This study addresses this gap by analyzing the incidence, management approaches, and outcomes of duodenal injuries treated at a military hospital in Sana’a, Yemen. By identifying the key factors influencing the success and failure of this study, our findings enhance evidence-based care for this vulnerable population and inform future clinical guidelines.

This article was posted as a preprint on Research Square on July 30, 2024.

## Materials and methods

Study design and setting

This retrospective observational study was conducted at a military hospital in Yemen. The study period was from June 2019 to December 2023. The hospital is the primary treatment center for trauma cases, particularly those arising from conflict-related injuries.

Study population

The study included patients of any age or sex who underwent exploratory laparotomy for abdominal trauma at the study center between June 2019 and December 2023 and had a confirmed diagnosis of duodenal injury during surgery and were classified using the American Association for the Surgery of Trauma (AAST) organ injury scale [9]. Only patients with complete medical records, including demographic information, injury characteristics, surgical details, and postoperative outcomes, were eligible. The exclusion criteria included patients with no intraoperative evidence of duodenal injury, those with incomplete or missing medical records, individuals with isolated duodenal injuries managed nonoperatively, and patients with significant pre-existing conditions unrelated to trauma that could confound the analysis.

Data collection

Data were retrospectively collected from hospital records to ensure comprehensive and accurate information. The collected variables included demographic data (age, sex), injury characteristics (mechanism of injury, such as penetrating or blunt trauma, Injury Severity Score (ISS), anatomical location of the duodenal injury, and associated injuries involving other organs). The details of surgical management were also documented, including the time from injury to operation, the type of surgical approach (exploratory laparotomy or damage control surgery), and the primary surgical technique used (e.g., primary closure, Roux-en-Y duodenojejunostomy, or primary repair with triple decompression). Outcome measures included postoperative complications, such as sepsis, chest-related complications, wound-related issues, and fistula formation, length of hospital stay, duration of intensive care unit (ICU) stay, mortality, and short-term success rate, defined as the absence of leaks or fistulas within 30 days postoperatively. To ensure data reliability, inconsistencies were cross-checked, and incomplete records were excluded from the analysis. This meticulous approach helped generate a robust dataset for evaluating duodenal injury management and outcomes.

Statistical analysis

Data were analyzed using IBM SPSS Statistics (v.25; IBM Corp., Armonk, NY). Descriptive statistics were used to summarize patient demographics, injury characteristics, and outcomes. Continuous variables were expressed as mean ± standard deviation (SD) or median (interquartile range, IQR), as appropriate. Categorical variables are presented as frequencies and percentages. Comparative analyses were performed to identify factors associated with successful versus unsuccessful outcomes. Chi-square tests were used for categorical variables, and independent samples t-tests were used for continuous variables. A p-value of <0.05 was considered statistically significant.

Ethical approval statement

This study was conducted in accordance with the ethical standards of the Declaration of Helsinki and local regulations governing medical research. Ethical approval for the study was obtained from the Ethics Committee of the General Military Hospital in Sana’a, Yemen. The study is a retrospective review of medical records for emergency surgical cases conducted between June 2019 and December 2023. Due to the retrospective nature of the study, the Ethics Committee waived the requirement for informed consent. Patient confidentiality was strictly maintained throughout the study, with all identifiable information anonymized during data collection and analysis. The ethical approval number for this study is IRB-MH-2024-005, and the study was approved on January 28, 2024.

## Results

A total of 520 exploratory laparotomy cases were reviewed during the study period (June 2019 to December 2022). Among these, 27 patients with confirmed duodenal injuries were identified and included in the analysis, with an incidence rate of 5.2%.

Demographic and injury characteristics

The cohort consisted predominantly of young males (n = 26, 96.3%), with a mean age of 21.93 ± 4.08 years. Most injuries were caused by penetrating trauma, including gunshot wounds (n = 12, 44.4%), explosive devices (n = 7, 25.9%), and shrapnel injuries (n = 7, 25.9%). The mean ISS was 20.63 ± 8.53, indicating moderate to severe injuries. Associated injuries were common, with colonic injuries observed in 73.9% (n = 17) of patients, followed by chest (n = 9, 39.1%) and liver (n = 8, 29.6%) injuries (Table 1).

**Table 1:** Demographic and injury characteristics. Abbreviations: SD, standard deviation; IQR, interquartile range; ISS, Injury Severity Score.

Characteristic	Frequency	Percentage
Age		
Mean age (± SD)	21.93 ± 4.075	
Median age (IQR)	22 (16-36)	
Age ≤18 years	5	18.5%
Age 19-30 years	21	77.8%
Age ≥31 years	1	3.7%
Gender		
Male	26	96.3%
Female	1	3.7%
Mechanism of injury		
Gunshot wound	12	44.4%
Explosive device	7	25.9%
Shrapnel	7	25.9%
Blunt injury	1	3.7%
ISS		
Minor (1-8)	2	7.4%
Moderate (9-15)	6	22.2%
Severe (16-24)	11	40.7%
Very severe (>25)	8	29.6%
Mean ISS (± SD)	20.63 ± 8.531	
Median ISS (IQR)	21 (6-38)	
Associated injuries		
Colonic injury	17	73.9%
Liver injury	8	29.6%
Chest injury	9	39.1%
Small bowel injury	6	26.1%
Stomach injury	3	13.0%
Kidney injury	4	17.4%
Head injury	3	13.0%
Vascular injury	2	7.4%
Diaphragm injury	2	8.7%
Gallbladder injury	1	4.3%
Pancreatic injury	1	3.7%
Ureteric injury	1	4.3%
Spleen injury	1	3.7%

Duodenal injury characteristics and management

The second segment of the duodenum (D2) was the most commonly affected segment, observed in 12 patients (44.4%). According to the AAST classification, 26 injuries (96.3%) were classified as grade II (laceration without ductal injury), and one injury (3.7%) was classified as grade III (laceration with ductal injury). The mean time from injury to surgery was 7.33 ± 3.61 hours. Surgical management primarily involved exploratory laparotomy in 22 patients (81.5%), while damage control surgery was performed in five patients (18.5%). The primary surgical techniques included primary closure in 18 patients (66.7%), primary repair with triple decompression in eight patients (29.6%), and Roux-en-Y duodenojejunostomy in one patient (3.7%) (Table 2).

**Table 2:** Duodenal injury characteristic (n = 27). Abbreviations: AAST, American Association for the Surgery of Trauma; SD, standard deviation; IQR, interquartile range.

Characteristic	Frequency	Percentage
Anatomical location		
D1–first part	6	22.2%
D2–second part	12	44.4%
D3–third part	5	18.5%
D4–fourth part	4	14.8%
Grade of injury (AAST scale)		
Grade II	26	96.3%
Grade III	1	3.7%
Surgical approach		
Exploratory laparotomy	22	81.5%
Damage control surgery	5	18.5%
Primary surgical technique		
Primary closure	18	66.7%
Primary repair with triple decompression	8	29.6%
Roux-en-Y duodenojejunostomy	1	3.7%
Time from injury to operation		
Mean time (hours ± SD)	7.33 ± 3.606	
Median time (IQR)	7 (4)	

Postoperative outcomes

Postoperative complications were observed in 19 out of 27 patients (70.4%), with sepsis being the most frequent complication, affecting 10 patients (52.6%). Chest-related complications occurred in nine patients (47.4%), while wound-related complications were noted in eight patients (42.1%). Fistula formation was reported in three patients (15.8%), and four patients (21.1%) required reoperation. Bile leaks and deep vein thrombosis (DVT) were each observed in two patients (10.5%), while disseminated intravascular coagulation (DIC) was noted in one patient (5.3%).

Despite the high complication rate, the short-term success rate, defined as the absence of leaks or fistulas at 30 days postoperatively, was achieved in 22 patients (81.5%). The mortality rate was three patients (11.1%), reflecting the severity of injuries within this study population.

The mean time to initiate enteral feeding was 5.89 ± 2.78 days. The average hospital stay was 26.52 ± 23.04 days, with a mean intensive care unit (ICU) stay of 6.74 ± 3.42 days, emphasizing the intensive care required for managing these injuries (Table 3).

**Table 3:** Postoperative complications and outcomes. Abbreviations: SD, standard deviation; IQR, interquartile range.

Characteristic	Frequency (n = 19)	Percentage (%)
Postoperative complications		
Sepsis	10	52.6
Chest-related complications	9	47.4
Wound-related complications	8	42.1
Fistula formation	3	15.8
Reoperation	4	21.1
Bile leak	2	10.5
Deep vein thrombosis (DVT)	2	10.5
Disseminated intravascular coagulation (DIC)	1	5.3
30-day postoperative outcomes		
Success rate	22	81.5
Mortality rate	3	11.1
Time from initiation of enteral feeding		
Mean time (days ± SD)	5.89 ± 2.78	
Length of hospital stay		
Mean length (days ± SD)	26.52 ± 23.04	
Duration of ICU stay		
Mean duration (days ± SD)	6.74 ± 3.42	

Risk factors influencing management outcomes

Key predictors of unsuccessful outcomes included shrapnel injuries, with four out of five unsuccessful cases (80.0%) having this mechanism compared to three out of 22 successful cases (13.6%) (χ²(1) = 9.343, p = 0.002; Fisher’s exact test, p = 0.009). A higher ISS was significantly associated with poorer outcomes, with unsuccessful cases having a mean ISS of 27.20 ± 9.34 compared to 19.14 ± 7.80 for successful cases (t(25) = 2.017, p = 0.05, Cohen’s d = 1.06). Grade III duodenal injuries were observed in one out of five unsuccessful cases (20.0%) compared to none in the successful cases (0.0%), showing a strong link to poorer outcomes (χ²(1) = 4.569, p = 0.033; Fisher’s exact test, p = 0.185). Additionally, patients managed with damage control surgery had a higher failure rate, with three out of five patients (60.0%) experiencing unsuccessful outcomes compared to two out of 22 patients (9.1%) undergoing exploratory laparotomy (χ²(1) = 6.998, p = 0.008; Fisher’s exact test, p = 0.030) (Table 4).

**Table 4:** Significant risk factors influencing management outcomes. Notes: The chi-square test or Fisher’s exact test was used for categorical variables. Effect size (φ, Phi) measures the strength of the association. An independent t-test was used for continuous variables. Cohen’s d measures the standardized difference between group means. Abbreviations: n: number of patients; %: percentage; χ²: chi-square statistic; p: p-value; t: t-value (t-test); ISS: Injury Severity Score; SD: standard deviation; φ: Phi coefficient (effect size for chi-square tests); d: Cohen’s d (effect size for t-tests); Ex. laparotomy: exploratory laparotomy; DCS: damage control surgery; N/A: not applicable.

Risk factor	Successful (n = 22)	Unsuccessful (n = 5)	Chi-square test	Fisher’s exact test	t-test	Effect size
Shrapnel injury	3 (13%)	4 (80%)	χ²(1) = 9.343, p = 0.002	p = 0.009	N/A	φ = -0.588 (large)
ISS (mean ± SD)	19.14 ± 7.803	27.20 ± 9.338	N/A	N/A	t(25) = 2.017, p = 0.05	d = 1.06 (large)
Grade of duodenal injury			χ²(1) = 4.569, p = 0.033	p = 0.185	N/A	φ = -0.411 (medium)
- Grade II	22 (100%)	4 (80%)				
- Grade III	0 (0.0%)	1 (20%)				
Surgical approach			χ²(1) = 6.998, p = 0.008	p = 0.030	N/A	φ = -0.509 (large)
Ex. laparotomy	20 (90.9%)	2 (40%)				
DCS	2 (9.1%)	3 (60%)				

## Discussion

This study aimed to evaluate the outcomes of duodenal injury management among patients with war trauma in Yemen, focusing on the incidence, severity, surgical approaches, postoperative complications, and factors influencing success or failure. Our findings were consistent with the existing literature and provided critical insights into the unique challenges and considerations of war trauma.

The incidence rate of duodenal injuries was 5.2% (n = 27) among 520 exploratory laparotomy cases, aligning well with reported incidence rates in the literature, ranging from 1% to 5% in cases of abdominal trauma [10]. This consistency underscores the reliability of our data in the context of global findings. Penetrating trauma was the predominant mechanism of injury, accounting for 95.7% (n = 26) of cases. This high prevalence of penetrating injuries, supported by similar findings in existing studies [4], highlights the significant impact of conflict and violence on trauma cases in our setting.

The study population predominantly consisted of young males (n = 26, 96.3%), with a mean age of 21.93 years, mirroring the demographics reported in previous studies. For instance, the National Trauma Data Bank reported a median age of 27 years for patients with duodenal trauma, with 80% of the patients being men [11]. This demographic profile is typical in conflict zones, where young men are more likely to be involved in violent encounters.

The ISS in our study had a mean value of 20.63 ± 8.53, indicating moderate to severe injuries. This result is comparable to those of other studies [12]. High ISS values indicate the severe nature of the injuries, which often involve multiple organ systems. The study revealed that most duodenal injuries were classified as AAST grade II (96.3%, n = 26), which is consistent with global patterns in which lower-grade injuries are more common [11]. Regarding anatomic location, injuries were most commonly found in the second duodenum part (D2), affecting 44.4% (n = 12) of cases, which is consistent with findings from other reviews [13]. This information is crucial for surgical planning and highlights the need for surgeons to be prepared for injuries at specific duodenal locations.

Associated injuries were frequent, with colonic injuries in 73.9% (n = 17), chest injuries in 39.1% (n = 9), and liver injuries in 29.6% (n = 8). These findings are in line with literature that emphasizes the high frequency of associated injuries due to the anatomical proximity of the duodenum to other vital organs [14,15]. The presence of multiple associated injuries complicates management and increases the risk of postoperative complications.

Postoperative complications were common, affecting 66.7% (n = 19) of patients. The most common complications were sepsis in 52.6% (n = 10), chest-related complications in 47.4% (n = 9), and wound-related complications in 42.1% (n = 8). These findings are consistent with existing literature, which reports high morbidity rates associated with duodenal injuries [12]. The high rate of sepsis underscores the need for vigilant postoperative care and early intervention to manage infections. One of the main complications of duodenal injuries is duodenal leaks, which can evolve into fistulas. The Memphis surgery group described a 19-year experience in managing these injuries and found that patients who developed duodenal leaks had longer hospital stays and higher rates of abdominal abscess formation [6]. Our findings agree with these observations, emphasizing the significant morbidity associated with duodenal leaks and the need for effective management strategies.

Several key risk factors influencing the outcomes of duodenal injury management were identified. Shrapnel injuries were particularly predictive of unsuccessful outcomes (p = 0.012). Higher ISS were significantly associated with poorer outcomes, consistent with other studies [5,7]. These findings highlight the importance of early and accurate assessment of injury severity to guide treatment decisions and improve outcomes.

The choice of surgical approach was a critical factor in our study. Exploratory laparotomy, performed in 81.5% (n = 22) of patients, was associated with better outcomes compared to damage control surgery, which was performed in 18.5% (n = 5) and linked to higher failure rates. This aligns with recent studies highlighting the critical role of damage control laparotomy (DCL) in trauma management. DCL is widely adopted in both military and civilian trauma settings for severely injured patients, with studies reporting high survivability and low rates of fecal diversion for hollow visceral injuries in military trauma [16]. While DCL is associated with increased resource utilization and higher complication rates, it does not significantly impact mortality rates in combat settings [17]. However, concerns about the overutilization of DCL have emerged, as it may expose patients to unnecessary morbidity and mortality [18]. Simplified surgical approaches focusing on damage control techniques for complex penetrating duodenal trauma have demonstrated improved survival and acceptable complication rates [4]. Complex procedures like Roux-en-Y duodenojejunostomy, performed in 3.7% (n = 1) of cases, remain associated with poorer outcomes, underscoring the need for careful surgical planning. The selection of the most appropriate technique should be guided by the severity and specific circumstances of each case to optimize patient outcomes.

Mortality in our study was 11.1% (n = 3), which is within the range of 3% to 24% reported in the literature [5,19]. This finding highlights the substantial risk of death associated with duodenal injuries, particularly in the context of war trauma. Early deaths were typically due to exsanguination from major vascular injuries, whereas late deaths were due to sepsis, duodenal fistula, and multiple organ failure. Factors such as associated pancreatic injuries, common bile duct injuries, and delayed injury recognition significantly increase mortality [20]. The high mortality rate associated with higher-grade injuries emphasizes the need for effective and timely management strategies to improve survival.

Our findings underscore the importance of early and accurate diagnosis of duodenal injuries to reduce treatment delays and improve outcomes. This requires the training of medical personnel in rapid assessment protocols, particularly in conflict zones where such injuries are prevalent. Additionally, the current study highlights the necessity of choosing an appropriate surgical approach based on injury severity and location. Primary repair for lower-grade injuries and complex repair for higher-grade injuries should be the standard practice to optimize patient outcomes. Moreover, the high incidence of postoperative complications, particularly sepsis and duodenal leaks, calls for enhanced postoperative monitoring and care protocols. Establishing standardized postoperative care routines can help mitigate these complications and improve patient recovery. Furthermore, our findings can inform the development of clinical guidelines and protocols for managing duodenal injuries in war zones. These protocols can standardize care, ensure treatment consistency, and ultimately improve patient outcomes across different settings. The current study also identified key areas for further research, including the development of advanced diagnostic tools, evaluation of surgical techniques, and exploration of novel postoperative care strategies. This approach can drive continuous improvement in the management of duodenal injuries.

Study limitations

Despite the valuable insights provided by our study, several limitations must be acknowledged. The retrospective study design limits the ability to establish causality, and as such, we cannot definitively conclude that damage control surgery directly contributes to poorer outcomes. Additionally, the relatively small sample size may limit the generalizability of the findings. The study was conducted at a single center in Yemen, which may not fully represent the experiences or practices of other regions or healthcare settings. There may also be issues related to the accuracy and completeness of the recorded data, which are inherent to retrospective reviews. Future studies should consider larger, multicenter designs and prospective methodologies to validate these findings and enhance their applicability across diverse settings.

## Conclusions

This study highlights the complexity of managing duodenal injuries in war trauma settings, with a high incidence of associated injuries and significant postoperative complications. Key factors influencing outcomes include injury severity, timely surgical intervention, and postoperative care. The findings emphasize the need for early diagnosis, appropriate surgical approaches, and vigilant postoperative care to improve patient outcomes. Future research should focus on prospective studies and multicenter analyses to validate these findings and enhance the generalizability of the results.

## References

[1] Rickard MJ, Brohi K, Bautz PC (2005). Pancreatic and duodenal injuries: keep it simple. ANZ J Surg.

[2] Khan MA, Garner J, Kelty C (2012). Management of duodenal injuries. Trauma.

[3] Leppäniemi A (2007). Focus on pancreatic and duodenal injuries. Eur J Trauma Emerg Surg.

[4] Ordoñez C, García A, Parra MW (2014). Complex penetrating duodenal injuries: less is better. J Trauma Acute Care Surg.

[5] Hong J, Wang SY, Qian L, Chen ZY (2015). Diagnosis and treatment of duodenal injury: a clinical analysis. Hepatogastroenterology.

[6] Schroeppel TJ, Saleem K, Sharpe JP (2016). Penetrating duodenal trauma: a 19-year experience. J Trauma Acute Care Surg.

[7] Ferrada P, Wolfe L, Duchesne J (2019). Management of duodenal trauma: a retrospective review from the Panamerican Trauma Society. J Trauma Acute Care Surg.

[8] Saleh A, Eissa M, Obadiel Y, Alsanabani A (2023). Early postoperative complications after exploratory laparotomy for abdominal trauma. Ain Shams J Surg.

[9] Coccolini F, Kobayashi L, Kluger Y (2019). Duodeno-pancreatic and extrahepatic biliary tree trauma: WSES-AAST guidelines. World J Emerg Surg.

[10] Saghafinia M, Nafissi N, Motamedi MR, Motamedi MH, Hashemzade M, Hayati Z, Panahi F (2010). Assessment and outcome of 496 penetrating gastrointestinal warfare injuries. J R Army Med Corps.

[11] Aiolfi A, Matsushima K, Chang G (2019). Surgical trends in the management of duodenal injury. J Gastrointest Surg.

[12] Velmahos GC, Constantinou C, Kasotakis G (2008). Safety of repair for severe duodenal injuries. World J Surg.

[13] Pandey S, Niranjan A, Mishra S, Agrawal T, Singhal BM, Prakash A, Attri PC (2011). Retrospective analysis of duodenal injuries: a comprehensive overview. Saudi J Gastroenterol.

[14] Huerta S, Bui T, Porral D, Lush S, Cinat M (2005). Predictors of morbidity and mortality in patients with traumatic duodenal injuries. Am Surg.

[15] Blocksom JM, Tyburski JG, Sohn RL (2004). Prognostic determinants in duodenal injuries. Am Surg.

[16] Smith IM, Beech ZK, Lundy JB, Bowley DM (2015). A prospective observational study of abdominal injury management in contemporary military operations: damage control laparotomy is associated with high survivability and low rates of fecal diversion. Ann Surg.

[17] Bograd B, Rodriguez C, Amdur R, Gage F, Elster E, Dunne J (2013). Use of damage control and the open abdomen in combat. Am Surg.

[18] Harvin JA, Wray CJ, Steward J (2016). Control the damage: morbidity and mortality after emergent trauma laparotomy. Am J Surg.

[19] Phillips B, Turco L, McDonald D, Mause A, Walters RW (2017). Penetrating injuries to the duodenum: an analysis of 879 patients from the National Trauma Data Bank, 2010 to 2014. J Trauma Acute Care Surg.

[20] Park YC, Kim HS, Kim DW (2022). Time from injury to initial operation may be the sole risk factor for postoperative leakage in AAST-OIS 2 and 3 traumatic duodenal injury: a retrospective cohort study. Medicina (Kaunas).

